# Typifications and synonymy in *Polystichum* (Dryopteridaceae) from Chile and Argentina

**DOI:** 10.3897/phytokeys.65.8620

**Published:** 2016-06-30

**Authors:** Rita E. Morero, David S. Barrington, Monique A. McHenry, João P. S. Condack, Gloria E. Barboza

**Affiliations:** 1 Departamento de Farmacia, Facultad de Ciencias Químicas, Universidad Nacional de Córdoba, (UNC), Av. Medina Allende y Haya de la Torre. Ciudad Universitaria, Córdoba. Argentina; 2 Instituto Multidisciplinario de Biología Vegetal (IMBIV-CONICET), Casilla de Correo 495, 5000 Córdoba; 3 University of Vermont, Pringle Herbarium, Torrey Hall, 27 Colchester Ave, Burlington, VT 05405; 4 Museu Nacional, Universidade Federal do Rio de Janeiro, Quinta da Boa Vista s.n., São Cristóvão, Rio de Janeiro, Brazil 20940-040

**Keywords:** Ferns, taxonomy, typification, nomenclature, South America

## Abstract

*Polystichum* Roth is one of the largest and most taxonomically challenging fern genera. South American species have a rich and complex nomenclatural history; many of the early names are inadequately typified. Based on extensive examination of original type material, we designate eleven lectotypes (including *Aspidium mohrioides*, Aspidium montevidense f. imbricata, Aspidium montevidense f. squamulosa, *Aspidium plicatum*, *Aspidium pycnolepis*, *Dicksonia andina*, *Polystichum elegans*, Polystichum mohrioides f. latifolia, Polystichum multifidum var. autranii, Polystichum platyphyllum var. kurtziana, and *Polypodium polystichoides*), and one neotype (*Polystichum brongniartianum*) for *Polystichum* taxa. Furthermore, three new synonyms are proposed.

## Abstract (es)

*Polystichum* Roth es uno de los géneros de helechos más grandes y de mayor complejidad taxonómica. Las especies sudamericanas tienen una rica y compleja historia nomenclatural, con muchos de los nombres inadecuadamente tipificados. En base a un minucioso examen del material tipo original, designamos lectotipos para once taxones de *Polystichum* (que incluyen *Aspidium mohrioides*, Aspidium montevidense f. imbricata, Aspidium montevidense f. squamulosa, *Aspidium plicatum*, *Aspidium pycnolepis*, *Dicksonia andina*, *Polystichum elegans*, Polystichum mohrioides f. latifolia, Polystichum multifidum var. autranii, Polystichum platyphyllum var. kurtziana y *Polypodium polystichoides*) y un neotipo (*Polystichum brongniartianum*). Además, se proponen 3 nuevos sinónimos.

## Introduction

*Polystichum* Roth (Dryopteridaceae) is a worldwide, taxonomically complex genus of ± 360–400 species (Zhang and Barrington 2013), characterized by highly variable species and convergent morphology (Kessler et al. 2005). Frequent hybridization, polyploidy (especially allopolyploidy), and apomixis hinder taxonomic delimitation of species (Little and Barrington 2003, Morero et al. 2015).

Taxa in the genus *Polystichum* can be recognized morphologically by stems usually ascending or erect, short internodes, and a dictyostelic stele. The monomorphic leaves bear several kinds of scales (hairs are rare); the ultimate segments are mostly asymmetric at base, often acroscopically auriculate, and ordinarily spinulose or at least dentate-mucronate at the margins. The sori are indusiate or not, when present the indusia are rounded-peltate.

South America, with ~40 species (43% of species in the New World), is an important world center of diversity (McHenry and Barrington 2014). The South American taxa have been studied largely using floristic and taxonomic approaches (Remy 1854, Fée 1869, 1873, Baker 1870, 1873, 1891, Hieronymus 1897, Christ 1905, Rosenstock 1906–1907, 1915, Hicken 1906, 1907a–b, 1909, Christensen 1910, Hosseus 1915, Looser 1939, 1968, Diem 1943, 1958, de la Sota 1977, Pichi-Sermolli and Bizarri 1978, Sehnem 1979, Smith 1985, Rodríguez Ríos 1987, Tryon and Stolze 1991, Marticorena and Rodríguez Ríos 1995, de la Sota et al. 1998, Kessler et al. 2005).

Recent work has revealed that the polystichums of Chile and Argentina pertain to two different monophyletic lineages, an exindusiate tropical Andean clade (Condack 2012, Condack et al. 2013, McHenry and Barrington 2014) and an indusiate austral South American clade (Morero et al. 2015, Barrington, unpublished data). A significant insight is that the tropical Andean taxa extend down the east face of the Andes and can reach the sea in southernmost Brazil and Uruguay (Condack 2012), whereas the austral Andean taxa are mainly confined to the subantartic region from 37° S to Cabo de Hornos (Morero et al. 2015).

In the course of work on revisions of *Polystichum* from Argentina and Chile we became aware that critical nomenclatural work with original materials was needed for a number of the taxa. In this manuscript we designate lectotypes for eleven taxa, one neotype, and propose three new synonyms for Central Andean and Southern Andean *Polystichum* which inhabits Argentina and Chile. We report the novelties for the two regions separately, alphabetically by basionym species name.

## Materials and methods

Review of types was based on examination of specimens in herbaria (BA, CONC, CORD, LIL, LP, SGO, SI; acronyms according to Thiers 2013), of digital images provided by source herbaria (B, BM, E, F, GENT, GH, GOET, K, LE, M, MPU, NY, P, S, US, W), or of digital images available via the JSTOR Global Plants portal (http://plants.jstor.org). All original protologues were reviewed.

Information about botanical publications, authors, dates, collectors and their herbarium and types, were taken from Stafleu and Cowan (1976–1988). The Melbourne Code (McNeill et al. 2012) was used for the proposed typification. The specimens selected as lectotypes are the best-preserved and most complete. The lectotype sheets are cited with the barcode number or indicated by the herbarium number, the former are cited with no space between the herbarium acronym and the number, while for accession numbers we have inserted a hyphen between the acronym and the number. The lectotype localities correspond to the geographical site mentioned on the specimen itself. If present, additional information (country and first subdivision) about collecting localities is indicated in square brackets. Photographs of the lectotypified specimens that are not available on JSTOR Global Plants are included here.

## Taxonomy

### Austral-Andean indusiate taxa

**1.** *Aspidium mohrioides* d´Urv., Flore des Iles Malouines: 26. 1825. Type: [Islas Malvinas] “I. Soledad”, n.d., *D. d’Urville 92.* Lectotype (designated here): P! (P00636426 [http://plants.jstor.org/stable/viewer/10.5555/al.ap.specimen.p00636426]). Syntypes remaining: [Islas Malvinas] “Iles Malouines, *D. d’Urville s.n.* – P! (P00636427 [https://science.mnhn.fr/institution/mnhn/collection/p/item/p00636427]). Iles Malouines, [year] 1815, *M. Lesson s.n.* – P! (P0036428 [https://science.mnhn.fr/institution/mnhn/collection/p/item/p00636428]).

= ***Polystichum mohrioides*** (d´Urv.) C.Presl, Tent. Pterid.: 83. 1836.

Three specimens are stored at P from Islas Malvinas, two collected by J. S. C. Dumont d´Urville (P00636426 and P00636427) and the third by R. P. Lesson (P00636428). The ferns were collected during a circumnavigation in the corvette “Le Coquille” (1822-1825) under the command of Captain L. Duperrey, in which Dumont d’Urville was a second officer and Lesson surgeon and naturalist (Duperrey 1828). Probably Dumont-d’Urville described *Aspidium mohrioides* based on these three specimens but he clearly cited “I. Soledad” as the collection site for the ferns collected in his voyage (Dumont-D´Urville 1825: 26). We designated P00636426 as the lectotype because it is the only one with the locality “I. Soledad”, and it is also the most complete sheet, consisting of a fertile plant and two additional fronds. The other two specimens comprise two (P00636428) or three (P00636427) mainly juvenile fronds.

**Figure 1.**
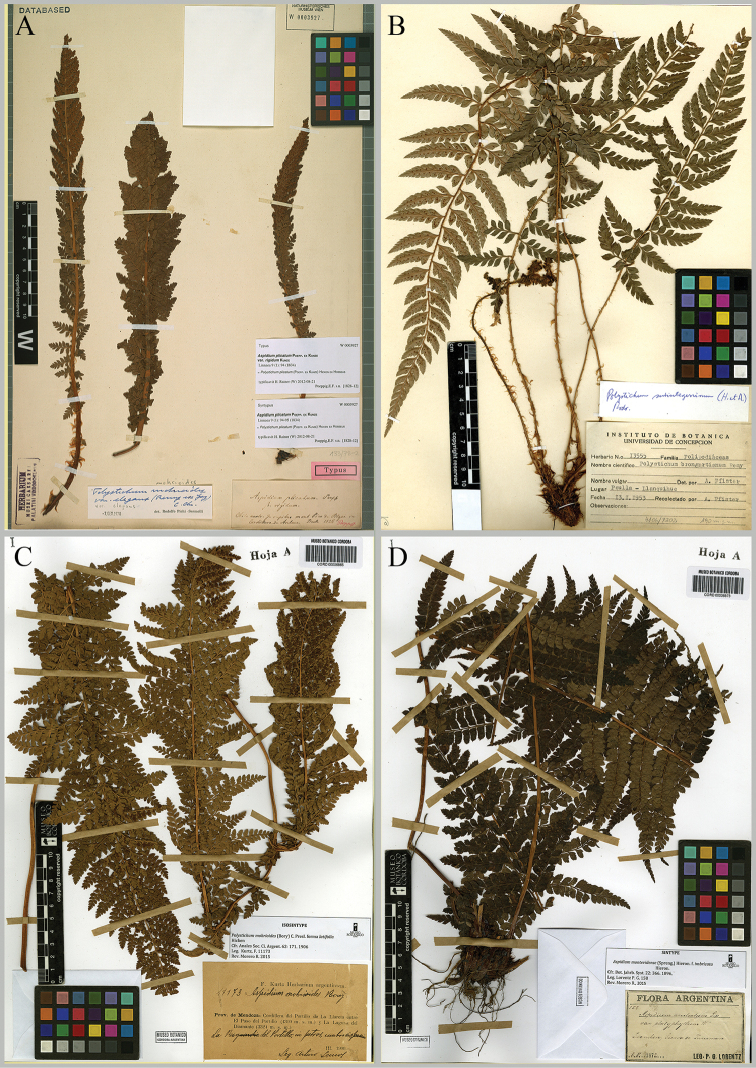
Lectotypes. **A** Lectotype of *Aspidium plicatum* (W) **B** Neotype of *Polystichum brongniartianum* (CONC) **C** Lectotype of Polystichum mohrioides f. latifolia (CORD) **D** Lectotype of Aspidium montevidense f. imbricata (CORD).

**2.** *Aspidium plicatum* Poepp. ex Kunze, Linnaea 9: 94. 1834.Type: [CHILE. Bio-Bio:] “Chile austr. in rupibus mont. Pico de Pilque in Cordillera de Antuco”, Dec 1828. *E. F. Poeppig* (*Diar. 745*). Lectotype (designated here): W! [W-0003927]; isolectotypes: LE! (LE00008146[http://plants.jstor.org/stable/viewer/10.5555/al.ap.specimen.le00008146], LE00008147 [http://plants.jstor.org/stable/viewer/10.5555/al.ap.specimen.le00008147], LE00008148 [http://plants.jstor.org/stable/viewer/10.5555/al.ap.specimen.le00008148]), P! (P00636429 [https://science.mnhn.fr/institution/mnhn/collection/p/item/p00636429], P00636430 [https://science.mnhn.fr/institution/mnhn/collection/p/item/p00636430], P00636431 [https://science.mnhn.fr/institution/mnhn/collection/p/item/p00636431]). Fig. 1A

= ***Polystichum plicatum*** (Poepp. ex Kunze) Hicken ex Hosseus, Trab. Inst. Bot. Farmacol. (Buenos Aires) 33: 9. 1915.

When Kunze (1834: 94) described *Aspidum plicatum*, no specimen referable as type was cited. At the same time, he recognized two named varieties (*α laxum* and *β rigidum*), citing one type specimen for each variety. According to the International Code of Nomenclature, Arts. 9.5 and 26.2 (McNeill et al. 2012), one lectotype for the species can be selected from the types of either of the two varieties. The features given in the protologue for var. *rigidum* closely match the description of *Aspidium plicatum*; for this reason, we select one sheet of the type collection of this variety as lectotype (W-0003927!). Among LE’s isolectotypes, there are two sheets (LE00008146! and LE00008148!), with incomplete data on their original labels; these labels state: *Aspidium plicatum* and *Aspidium plicatum β* respectively, being both Poeppig’s gatherings; it is supposed that they are also duplicates.

**3.** *Dicksonia andina* Phil., Anales Univ. Chile 94: 359. 1896. Type: [CHILE. Valdivia:] “Cuesta de Lipela, Cord. de Ranco”, Jan 1887, *O. Philippi s.n.* Lectotype [designated here (second step lectotypification after Rodríguez Ríos 1987: 51)]: SGO! (SGO000000511 [http://plants.jstor.org/stable/10.5555/al.ap.specimen.sgo000000511]); isolectotype: SGO! (SGO000000512 [http://plants.jstor.org/stable/10.5555/al.ap.specimen.sgo000000512]). Syntypes remaining: [CHILE. Valdivia:] “ad flumen Palena”, Jan-Feb 1887, *A. Hirth s.n.* – SGO! (SGO 000000513 [http://plants.jstor.org/stable/10.5555/al.ap.specimen.sgo000000513]). [CHILE. Valdivia:] “Prope flumen Manso raram leg. Dr. Reiche” (not found).

= ***Polystichum multifidum*** (Mett.) T. Moore, Index Fil. (T. Moore): 84. 1857. Basyonym: *Aspidium multifidum* Mett., Fil. Lechl. 1: 20. 1856.

Rudolph A. Philippi misapplied the name *Dicksonia* (Fam. Dicksoniaceae), a genus of tree ferns, to specimens of *Polystichum*. We found three sheets at SGO. Of these, two were collected by his grandson Otto Philippi and the third one by Hirth. The original Philippi label of SGO000000513!, indicates “*F. Hirth*” as the collector name in error, the correct name is “Ad. [Adolfo] Hirth”, as it is written in the protologue. Both accessions of O. Philippi’s collection are well preserved. Rodríguez Ríos (1987) unintentionally lectotypified a Philippi´s specimen (“SGO-isosyntypus!”). As in SGO exists two sheets of Philippi, a second step lectotypification is here proposed in accordance to Art. 9.17. The selected lectotype (SGO000000511!) is the most complete sheet including one leaf (with intact lamina and petiole).

**4.** *Polystichum brongniartianum* J.Rémy, in Fl. Chil. [Gay] 6: 518. 1854. Type: [CHILE. Concepción]: “Chile Austral, Concepción” *C. Gay* s.n., not localized. Neotype (designated here): CHILE. Llanquihue: Peulla. 13 Jun 1953, *A. Pfister s.n.* CONC! (CONC-13555). Fig. 1B

= ***Polystichum subintegerrimum*** (Hook. & Arn.) R.Rodr., Gayana. Bot. 44: 48. 1987. Basyonym: *Aspidium subintegerrimum* Hook. & Arn., Bot. Beechey Voy.: 52. 1832.

The type specimen of *Polystichum brongniartianum* (C. Gay s. n.) cited by Rémy (1854), is supposed to be housed at P; after a careful search in P, we have found no type material of this name. Neither was any found by other fern taxonomists in other herbaria (Christensen 1910, Looser 1968, Rodríguez Ríos 1987). We propose CONC-13555 as neotype, as it fits Rémy’s description in its narrowest interpretation.

**5.** *Polystichum elegans* J.Rémy, in Fl. Chil. [Gay] 6: 514. 1854. Type: [CHILE. Prov. Colchagua: “Cordillera de Talcaregué, pie le Volcán de Cordier”, Feb 1831, *C. Gay* 29. Lectotype [designated here (second step lectotypification after Rodríguez Ríos 1987: 49)]: P! (P00636434 [http://plants.jstor.org/stable/10.5555/al.ap.specimen.p00636434?searchUri=filter%3Dname%26so%3Dps_group_by_genus_species%2Basc%26Query%3Dpolystichum%2Belegans]); isolectotypes: P! (P00636432 [https://science.mnhn.fr/institution/mnhn/collection/p/item/p00636432], P00636435 [https://science.mnhn.fr/institution/mnhn/collection/p/item/p00636435]).

= ***Polystichum plicatum*** (Poepp. ex Kunze) Hicken ex Hosseus, Trab. Inst. Bot. Farmacol. (Buenos Aires) 33: 9. 1915. Basyonym: *Aspidium plicatum* Poepp. ex Kunze, Linnaea 9: 94. 1834.

There are three sheets of Gay at P, two sheets are housed as Gay 29 (P00636434! and P00636432!) with data of the collection place, while the third sheet (P00636435!) lacks collector number and only “Chili” is indicated as the collection location. Rodríguez Ríos (1987) wrote “Se halla en las altas cordilleras de Talcaregue, provincia de Colchagua, en las orillas de los arroyos, cerca del volcán en donde es algo rara (P!)”. The Gay 29’s collection has a label with the same information cited by Rodríguez Ríos. As a second step lectotypification is necessary, we select P00636434 as the second step lectotype because it includes the diagnostic character for this taxon and it is the most complete accession (three fronds and the petiole scales are intact).

**6.** Polystichum mohrioides (d´Urv.) C. Presl f. latifolia Hicken, Anales Soc. Ci. Argent. 62: 171. 1906. Type: [ARGENTINA. Mendoza. Dpto. San Carlos:] “Cordillera del Portillo de La Llareta entre El Paso del Portillo (4300 m.s.m.) y la Laguna del Diamante (3324 m.s.m.). La Resguardia del Portillo, in petros, umbrosis passim. Leg. Arturo Lemos”, Mar 1900, *F. Kurtz 11173.* Lectotype (designated here): CORD! (CORD00006865 Hoja A); isolectotypes: CORD! (CORD00006866 Hoja B), SI! (SI000111). Syntypes remaining: [ARGENTINA. Tierra del Fuego:] “Canal Beagle. Punta Remolino, 1300 m.” Mar 1903, *M. S. Pennington 422* – SI! (SI-60478).[ARGENTINA] “Prov. Neuquén. Departam. Huiliches. Junín de los Andes, entre Fortín Maipú y Laguna Lolog” 13-14, IV, 1888, *F. Kurtz 6391*– CORD! (CORD00006867 Hoja A, CORD00006869 Hoja B, CORD00006884). Fig. 1C.

= ***Polystichum plicatum*** (Poepp. ex Kunze) Hicken ex Hosseus, Trab. Inst. Bot. Farmacol. (Buenos Aires) 33: 9. 1915. Basyonym: *Aspidium plicatum* Poepp. ex Kunze, Linnaea 9: 94. 1834.

We examined the three syntypes of Polystichum mohrioides f. latifolia. All three are of good quality; two are preserved at CORD, and the third at SI. One of the two sheets of Kurtz 11173 (leg. A. Lemos) at CORD is designated as lectotype: CORD 00006865! Hoja A. This accession consists of three mature fertile fronds versus Hoja B (isolectotype) with all three sterile leaves.

**7.** Polystichum multifidum (Mett.) T.Moore var. autranii Hicken, Anales Soc. Ci. Argent. 62: 172. 1906. Type: [ARGENTINA] “Chubut: Laguna Blanca, *J. Koslowsky n° 240*”. Lectotype (designated here): SI000112!; isolectotypes: SI000113!

= ***Polystichum multifidum*** (Mett.) T. Moore, Index Fil. (T. Moore): 84. 1857. Basyonym: *Aspidium multifidum* Mett., Fil. Lechl. 1: 20. 1856.

Although there are two sheets of this variety at SI, each indicated as holotype and isotype respectively, it is not possible to be sure that it is from the protologue. Holotype and isotype labels have been added by the SI herbarium staff after Hicken’s death. We select as lectotype, SI000112 sheet since it has a label with detailed collection data and consists of two complete fronds.

**8.** Polystichum multifidum (Mett.) T.Moore var. dusenii C.Chr., Ark. Bot. 10 (2): 19. 1910. syn. nov. Type: [CHILE, Aysén:] “Patagonia Occ. in valle fluminis Aysén”, 15 Jan 1897, *P. Dusén* 489 Holotype: S! (S05-10906 [http://plants.jstor.org/stable/10.5555/al.ap.specimen.s05-10906])

= *Polystichum chilense* (H.Christ) Diels, Nat. Pflanzenfam. [Engler & Prantl] 1: 192. 1899. Basyonym: *Aspidium aculeatum* var. *chilense* (H.Christ) Ber. Schweiz. Bot. Ges. 3: 39. 1893.

Christensen (1910) recognized Polystichum multifidum var. variety based on lamina division (twice pinnate-pinnatifid lamina), and shape and margin of pinnules (pinnules obovate with slightly dentate margins). Looser (1968) suggested that this set of plants should be considered a variety of *Polystichum chilense*; later, Rodríguez Ríos (1987) agreed and added another diagnostic character (coriaceous pinnules), to support this position and formalized the recognition of the variety: Polystichum chilense var. variety (C.Chr.) R.Rodr. We observed frequent variation in the lamina division among *Polystichum chilense* populations, even within the same population. This variability is often associated with the size and age of the plant (Barrington 2012). Consequently, we synonymize var. *dusenii* under *Polystichum chilense*.

9. *Polystichum pearcei* Phil., Linnaea 33: 305. 1865. syn. nov. Type. [CHILE] Valdivia, Cordillera de Ranco, s.f., *R. Pearce* s.n. Holotype: SGO! (SGO000000478 [http://plants.jstor.org /stable/10.5555/al.ap.specimen.sgo000000478])

= *Polystichum multifidum* (Mett.) T. Moore, Index Fil. (T. Moore): 84. 1857. Basyonym: *Aspidium multifidum* Mett., Fil. Lechl. 1: 20. 1856.

The main diagnostic characters for *Polystichum pearcei* are the 3-pinnate frond and the rachis slightly scaly (Philippi 1865). Rodríguez Ríos (1987) considered the same diagnostic characters but subordinated this species as a variety under *Polystichum multifidum*. Analyzing herbarium specimens and field observations of *Polystichum multifidum*, we found high variability in the lamina division and density of scales on the rachis. In addition, when *Polystichum multifidum* grows in warmer and/or drier conditions, the leaves are smaller, less divided, and not as scaly (Morero, pers. obs.). Since the type of *Polystichum pearcei* falls within the variation of *Polystichum multifidum*, we propose it as a synonym. Molecular studies in progress support this proposal (Barrington pers. comm.).

### Tropical Andean exindusiate taxa

**10.** Aspidium montevidense (Spreng.) Hieron. f. imbricata Hieron., Bot. Jahrb. Syst. 22 (3): 366. 1897. Type: [ARGENTINA, Tucumán:] “Siambón, Sierra de Tucumán”, 8 May 1872, *P. G. Lorentz 158*. Lectotype (designated here): CORD! (CORD00006873); isolectotypes: CORD! (CORD00006874!), SI! (SI-088094), US! (US00067142 [http://plants.jstor.org/stable/10.5555/al.ap.specimen.us00067142]). Syntype remaining: [ARGENTINA, Tucumán:] “En las pendientes y las quebradas de la cuesta de Garabatal, cerca de Siambón, Sierra de Tucumán”, 27 Jan 1874, *P. G. Lorentz & G. Hieronymus 801* – (isosyntype: CORD! [CORD00006875]). Fig. 1D.

= ***Polystichum montevidense*** (Spreng.) Rosenst., Hedwigia: 46: 111, 1906. Basyonym: *Polypodium montevidense* Spreng., Syst. Veg. (ed. 16) [Sprengel] 4 (1): 59. 1827.

This name was based on two syntypes both from Tucumán hills (Argentina): *Lorentz 158* and *Lorentz & Hieronymus 801*. Hieronymus described this taxon with the specimens deposited at B, but a careful search of B does not yield them. Four duplicates of *Lorentz 158* exist in herbaria: two housed at CORD, one at US, and a fourth at SI. We select CORD00006873 as the lectotype because it represents an entire plant including a rhizome, fertile fronds with petiole base and rhizome born intact. Characters used to diagnose the species by Condack et al. (2013)—rhizome and petiole scale color, shape, and margin along with the shape and margin of the pinnules—are present in our selected lectotype.

**Figure 2.**
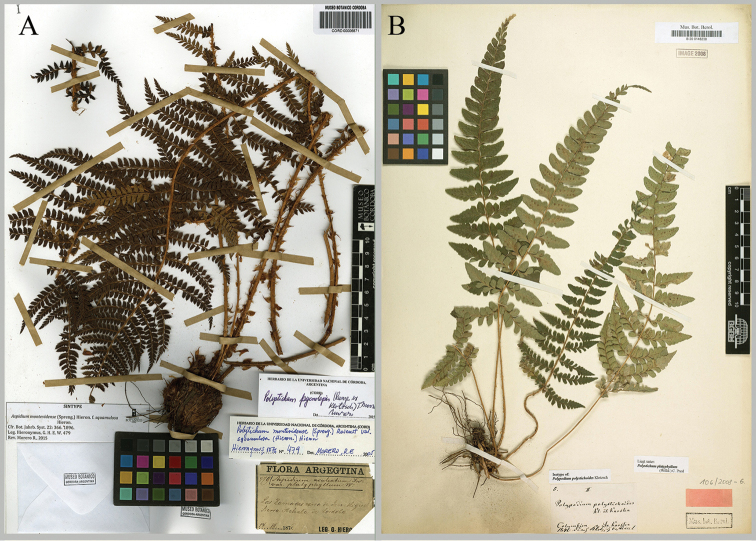
Lectotypes. **A** Lectotype of Aspidium montevidense f. squamulosa (CORD) **B** Lectotype of *Polypodium polystichoides* (B).

**11.** Aspidium montevidense (Spreng.) Hieron. f. squamulosa Hieron., Bot. Jahrb. Syst. 22 (3): 366. 1897. Type: [ARGENTINA, Córdoba:] “Las Ramadas cerca de San Miguel, Sierra Achala de Córdoba”, 14 Mar 1876, *G. Hieronymus 479.* Lectotype (designated here): CORD! (CORD00006871); isolectotypes: CORD (CORD00006887]), F! (F00075611 [http://plants.jstor.org /stable/10.5555/al.ap.specimen.f0075611f]), SI! (SI-088100). Syntypes remaining: [ARGENTINA, Córdoba:] “Sierra de Achala, cerca del Puerto Alegre”, 5 Feb 1877, *G. Hieronymus 805* – CORD! (CORD00006870, CORD00006886), F! (F0075612 [http://plants.jstor.org/stable/10.5555/al.ap.specimen.f0075612f]), US! (US00067143! [http://plants.jstor.org/stable/10.5555/al.ap.specimen.us00067143]). [ARGENTINA, Córdoba:] “mit der hauptform in einer Schlucht am westlichen Fuβ der Gigantes (*G. Hieronymus s.n.* 2 Feb 1883)”, (not found). Fig. 2A.

= ***Polystichum pycnolepis*** (Kunze ex Klotzsch) Hieron., Bot. Jahrb. Syst. 34 (4): 452. 1904. Basyonym: *Aspidium pycnolepis* Kunze ex Klotzsch, Linnaea 20: 365. 1847.

We located two of the three syntypes cited by Hieronymus (1897). Both are high-quality specimens in agreement with the diagnosis. We have selected Hieronymus 479 at CORD as the lectotype because it is a more complete specimen including all the diagnostic characters for this taxon (the rhizome scales are intact and there are two fronds with fragments of petioles and blades). After a careful analysis of morphological characters of type specimens we have found that their identifying characters (large petiole scales bicolorous with the center atropurpureous and the edge rufous; pinnules coriaceous and revolute with margins serrate and spinules well developed), match fairly the diagnostic characters of *Polystichum pycnolepis*. Therefore, we confirm the synonymy of this form under *Polystichum pycnolepis*, as proposed by Condack et al. (2013).

**12.** *Aspidium pycnolepis* Kunze ex Klotzsch, Linnaea 20: 365. 1847. Type: [VENEZUELA] “Columbia, Col. Tovar”. 1856. *J. W. K. Moritz 296 b.* Lectotype (designated here): B! (B200148046-b [http://ww2.bgbm.org/herbarium/specimen.cfm?SpecimenPK=83712&idThumb=279937&SpecimenSequenz=1&loan=0]); isolectotypes: B! (B200148045-b [http://ww2.bgbm.org/herbarium /specimen.cfm?SpecimenPK=166846&idThumb=279936&SpecimenSequenz=1&loan=0]); K! (K000512992 [http://apps.kew.org/herbcat/detailsQuery.do?imageId=231125&pageCode=1&presentPage=1&queryId=2&sessionId=E0DC715CEF51CFBB8FB85F938E0D6780&barcode=K000512992]. Syntypes remaining: [VENEZUELA] “Columbia”. 1856. *J. W. K. Moritz 296* – B! (B200148177 [http://ww2.bgbm.org/herbarium/specimen.cfm?SpecimenPK=83714&idThumb=279972&SpecimenSequenz=1&loan=0], B200148176 [http://ww2.bgbm.org/herbarium/specimen.cfm?SpecimenPK=83713&idThumb=279971&SpecimenSequenz=1&loan=0]); K! (K000512989 [http://apps.kew.org/herbcat/detailsQuery.do?imageId=231122&pageCode=1&presentPage=1&queryId=1&sessionId=E0DC715CEF51CFBB8FB85F938E0D6780&barcode=K000512989]), M! (not available on line), NY! (not available on line). [VENEZUELA] “Columbia”. 1856. *J. W. K. Moritz 295* – B! (B200127536 [http://ww2.bgbm.org/herbarium/specimen.cfm?SpecimenPK=72797&idThumb=265393&SpecimenSequenz=1&loan=0], B200148175 [http://ww2.bgbm.org/herbarium/specimen.cfm?SpecimenPK=83711&idThumb=279970&SpecimenSequenz=1&loan=0]); M! (not available on line); P! (P00604457 [https://science.mnhn.fr/institution/mnhn/collection/p/item/p00604457?listIndex=4&listCount=30]); *J. W. K. Moritz s.n.* K! (K000512992[http://apps.kew.org/herbcat/detailsQuery.do?imageId=231125&pageCode=1&presentPage=1&queryId=3&sessionId=E0DC715CEF51CFBB8FB85F938E0D6780&barcode=K000512992]). [VENEZUELA] “Columbia”, *G. K. W. Karsten Coll. II. a.* B! – (B200148170 [http://ww2.bgbm.org/herbarium/specimen.cfm?SpecimenPK=83716&idThumb=279969&SpecimenSequenz=1&loan=0]).

= ***Polystichum pycnolepis*** (Kunze ex Klotzsch) Hieron., Bot. Jahrb. Syst. 34 (4): 452. 1904.

The type material of *Aspidium pycnolepis* is a mixed collection. In order to stabilize the application of this name in the sense that it has been used by most other botanists, we choose *Moritz 296 b* (B200148046!) since most closely represents the original description. The remaining syntypes, after the designation of the lectotype of *Aspidium pycnolepis*, pertain to at least three species, only one of which is *Polystichum pycnolepis*. We have seen the following: “Columbia, Páramo de la Culata”, *Moritz 296* (B! [det. *Polystichum orbiculatum* and *Polystichum gelidum*, see annotation labels]; K! [det. *Polystichum gelidum*]; M! [det. *Polystichum pycnolepis*]; NY! [det. *Polystichum pycnolepis*]; P-photo! [det. *Polystichum gelidum*]); same locality *Moritz 295* (B! [det. *Polystichum pycnolepis* and *Polystichum orbiculatum*, see annotation labels]; M! [det. *Polystichum pycnolepis*]); *Moritz s.n.* – K! [det. *Polystichum pycnolepis*].

**13.** *Polypodium polystichoides* Klotzsch, Linnaea 20: 383, 1847, **syn. nov.** Type: [COLOMBIA] “Columbien 1846 (Col. II)”, s.d. *H. Karsten* 5. Lectotype (designated here): B! (B200148238 [http://herbarium.bgbm.org/object/B200148238]); isolectotypes: B! (B200148237 [http://herbarium.bgbm.org/object/B200148237], B200148239 [http://herbarium.bgbm.org/object/B200148239]); LE (not found). Fig. 2B.

= ***Polystichum platyphyllum*** (Willd.) C.Presl, Tent. Pterid. 84. 1836. Basyonym: *Aspidium platyphyllum*, Sp. Pl. ed. 4, 5 (1): 255. 1810.

There are three sheets of Karsten at B (one more, not found, may be housed at LE); from among these, we select B200148238 as the lectotype, because it is a more complete accession containing an entire plant. Based on a careful observation of the three specimens, we consider that all pertain to *Polystichum platyphyllum* — by the lamina dissection, the shape and color of petiole scales, and the elongate once-pinnate and bulbil-bearing lamina apex— which are diagnostic characters of this taxon.

**14**. Polystichum platyphyllum (Willd.) C.Presl var. kurtziana Hicken, Anales Soc. Ci. Argent. 63: 169. 1907. Type: [ARGENTINA] “Prov. Córdoba: Departam. San Alberto. Sierra Grande: Cuesta del Tránsito: Región del Tabaquillo. An feuchten Orten in grossen Gruppen; zerstreut”, 6-13 Jan 1895, *F. Kurtz 8352.* Lectotype (designated here): SI! (SI000116 [http://plants.jstor.org/stable/10.5555/al.ap.specimen.si000116]); isolectotypes: CORD! (CORD00006736 Hoja A; CORD00006737 Hoja B), NY! (NY00149457 [http://plants.jstor.org/stable/10.5555/al.ap.specimen.ny00149457]).

= ***Polystichum montevidense*** (Spreng.) Rosenst., Hedwigia: 46: 111. 1906. Basyonym: *Polypodium montevidense* Spreng., Syst. Veg. (ed. 16) [Sprengel] 4(1): 59. 1827.

A single collection was cited in the protologue of this varietal name. Of the four duplicates of the type collection, two are housed at CORD, one at NY and the fourth at SI. The last one, with a Hicken’s handwritten label with the inscription “au var? nova?”, was supposedly used by this author for the diagnosis. According to the recommendation of the International Code of Nomenclature (Rec. 9A.3, McNeill et al. 2012), the specimen with an author’s annotations on herbarium sheets should be given preference in choosing the lectotype; therefore, SI000116 is designated here as the lectotype.
